# Chemically Anchored Diamond with H3 Centers for Ratiometric Measurement of Isolated Mitochondria Temperature

**DOI:** 10.3390/ijms262311395

**Published:** 2025-11-25

**Authors:** Alexey M. Romshin, Alexey G. Kruglov, Anna B. Nikiforova, Alexander A. Zhivopistsev, Rustem H. Bagramov, Vitaly I. Korepanov, Dmitrii G. Pasternak, Yuri M. Shlyapnikov, Timur M. Valitov, Vladimir P. Filonenko, Igor I. Vlasov

**Affiliations:** 1 Prokhorov General Physics Institute of the Russian Academy of Sciences, 119991 Moscow, Russia; azh253@gmail.com (A.A.Z.); dg.pasternak@physics.msu.ru (D.G.P.); timur.valitov25@gmail.com (T.M.V.); vlasov@nsc.gpi.ru (I.I.V.); 2 Institute of Theoretical and Experimental Biophysics of the Russian Academy of Sciences, 142290 Pushchino, Russia; nikiforanna888@gmail.com (A.B.N.); yuri.shlyapnikov@gmail.com (Y.M.S.); 3 Vereshchagin Institute of High Pressure Physics of the Russian Academy of Sciences, 108840 Moscow, Russia; bagramov@mail.ru (R.H.B.); vpfil@mail.ru (V.P.F.); 4 Institute of Microelectronics Technology and High-Purity Materials of the Russian Academy of Sciences, 142432 Chernogolovka, Russia; korepanov@iptm.ru; 5 Physics Faculty, M.V. Lomonosov Moscow State University, 119991 Moscow, Russia

**Keywords:** diamond thermometer, mitochondria, cell thermodynamics, color centers

## Abstract

Precise measurement of mitochondrial temperature at different metabolic states remains one of the key challenges in cellular biophysics due to the lack of thermometers that combine nanoscale sensitivity with stable thermal contact with the organelle. Here, we present a hybrid sensing platform based on chemically functionalized diamond microparticles containing H3 luminescent centers, covalently bound to the outer membrane of isolated rat liver mitochondria. Surface activation via oxidation and EDC/HOBt chemistry provides a robust and reproducible thermal link between the thermometric probe and the organelle, minimizing heat dissipation through the surrounding medium. The local temperature is monitored ratiometrically from the emission ratio of H3 centers at 515–525 nm and 585–610 nm, showing a linear dependence on temperature with a relative sensitivity of 1.15%⋅°C^−1^ in aqueous environments. Upon the uncoupling of oxidative phosphorylation and the inhibition of electron transport, the diamond thermometers reproducibly recorded the local thermal changes in the range of 0.5–10 °C, depending on the degree of coverage by anchored mitochondria. The observed response reflects efficient local heat confinement within the diamond–mitochondrion assembly, suggesting that structural organization and thermal insulation at the subcellular level are critical modulators of mitochondrial thermogenesis.

## 1. Introduction

Mitochondria play a key role in the regulation of intracellular homeostasis, coupling anabolic and catabolic pathways and serving as the primary suppliers of ATP in the cell [1]. It is also known that between 40% and 60% of the energy released during electron transfer along the respiratory chain in oxidative phosphorylation is dissipated as heat, making mitochondria important thermogenic organelles, especially under conditions of high energy demand and during the uncoupling of respiration and oxidative phosphorylation [2]. In specialized tissues, such as brown adipose tissue, thermogenesis appears to be the primary mitochondrial function, contributing to the maintenance of body temperature through the uncoupling of oxidative phosphorylation by the specific proton-conducting uncoupling protein UCP1 [3]. In recent years, experimental data have indicated the existence of subcellular temperature gradients and even the potential role of temperature as a signaling parameter—a phenomenon referred to as “thermal signaling” [4].

In this context, mitochondria function not only as metabolic hubs but also as thermodynamic centers of the cell, capable of generating and modulating the local thermal field. However, the experimental detection of such temperature heterogeneity remains a methodologically challenging task [5].

Optical methods employing temperature-sensitive fluorophores have become the most widely used approach for measuring local temperatures in cellular compartments. These include molecular dyes (e.g., Mito and ER Thermo Yellow [6,7,8], Rhodamine B-derivatives [9,10]), fluorescent proteins (tsGFP, gTEMP [11,12,13]), polymeric nanogels [14,15,16,17,18], rare-earth complexes (e.g., Eu-TTA [19,20,21]), quantum dots [22,23,24,25], and diamond nanoparticles with color centers [26,27,28,29,30]. The listed sensors differ in sensitivity, photostability, size, targeted delivery capability, and dependence on external factors such as pH, viscosity, ionic strength, oxygen availability, redox state, and membrane potential. These variations result in a wide range (up to several tens of degrees Celsius) in the measured local temperatures, particularly in mitochondria under identical conditions [5]. Thus, despite the abundance of studies in recent years, a definitive conclusion regarding the existence of a significant temperature gradient between mitochondria and other cellular compartments remains elusive.

At the same time, the use of micro- and nanodiamonds, whose luminescence is sensitive to temperature—including within the physiological range—appears to be a promising approach for the precise registration of ultralocal temperatures in cells and cellular organelles [27,28,29,30,31,32,33,34]. Compared to other approaches, the use of diamond particles offers several advantages, including the lack of dependence of their luminescence spectral properties on the microenvironment and their exceptional temporal stability.

However, despite these advantages, the use of diamond nanoparticles—as well as any other free nanoparticles—in systems with isolated mitochondria or in cell culture is associated with another source of artifacts: the degree of contact between the sensor and the heat source. The absence of a stable and tight contact introduces an additional channel for heat dissipation into the medium filling the space between the sensor and the source. As a result, the thermal resistance between them increases, causing the measured value to reflect not the true mitochondrial temperature but rather an average value along the heat source’s thermal profile—lower in absolute terms. Variations in the thickness of the aqueous layer, arising from the diffusive movement of free particles and mitochondria in suspension, can lead to instability in temperature readings. We suggest that poorly controlled thermal contact was the source of the substantial variation in the mitochondrial temperature values we previously measured using microdiamonds with SiV centers [30].

Thus, solving the problem of establishing a stable contact between the thermometer and mitochondria, as well as increasing the proportion of the surface in contact with mitochondria, should enable accurate measurements of temperature changes in mitochondria under various functional states. In the present study, we propose a universal strategy for establishing a reliable thermal contact between a diamond thermometer and mitochondria. This strategy is based on chemical activation of the probe surface, which ensures strong attachment of the thermometer to the organelle membrane and minimal thermal resistance. Using this approach, we demonstrate for the first time temperature changes in isolated mitochondria during respiratory uncoupling and inhibition of electron transport, as detected through the ratiometric luminescence response of H3 color centers in micron-sized diamonds.

## 2. Results

**Figure 1:**
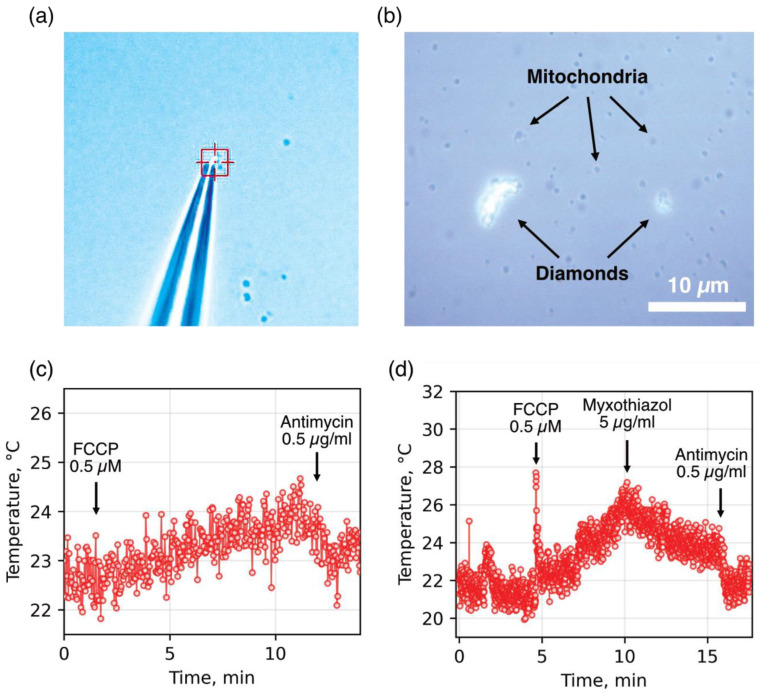
Measurement of the temperature of isolated mitochondria using (**a**) fixed and (**b**) dispersed diamonds in suspension. Mitochondria (0.1 and 0.2 mg protein/mL) were placed in a sucrose–mannitol-based medium supplemented with 5 mM glutamate and 5 mM malate. Then, using a micromanipulator under microscopic control, an individual mitochondrion was gently pressed onto the slide using a diamond attached to the tip of a glass micropipette (red box) (**a**). Alternatively, diamond particles were dispersed into the mitochondrial suspension and allowed to spontaneously aggregate for ~5–6 min (**b**). Temperature changes were determined by monitoring the shift in the zero-phonon line (ZPL) of SiV-center luminescence. (**c**,**d**) Corresponding temperature traces. Arrows indicate the addition of the uncoupler FCCP and respiratory chain inhibitors (myxothiazol and antimycin A). The brief temperature spikes observed immediately after FCCP addition in panel (**d**) are attributed to heat release caused by the exothermic reaction associated with the positive enthalpy of mixing of alcohol and aqueous medium. Representative traces from over 50 independent recordings are shown.

In the first stage, to improve the stability and area of thermal contact between the sensor and the heat source, we tested two approaches using diamond microparticles containing SiV centers with native surfaces. The first approach involved pressing a mitochondrion against a glass slide using a diamond particle fixed to the tip of a glass micropipette (Figure 1a; see [30] for details). This significantly improved the stability of the diamond–mitochondrion thermal contact but did not resolve the issue of the low ratio between the sensor’s contact area and that of other heat-absorbing media and surfaces. As a result, the measured mitochondrial temperature fluctuations in the presence of an oxidative phosphorylation uncoupler and respiratory chain inhibitors remained within a relatively narrow range (Figure 1c). Moreover, the mechanical pressure exerted by the diamond particle on the mitochondria could lead to damage and loss of functional properties.

The second approach involved spontaneous aggregation of diamond sensors with dynamic mitochondrial clusters (Figure 1b), which, under favorable conditions, resulted in a significantly broader range of temperature changes compared to individual mitochondria (Figure 1d). However, the low stability of diamond–mitochondrion associations, along with the Brownian motion of organelles and diamonds, often led to detachment of aggregates from the thermometer upon the addition of substrates, uncouplers, and inhibitors, thereby reducing the reproducibility of the obtained results.

**Figure 2:**
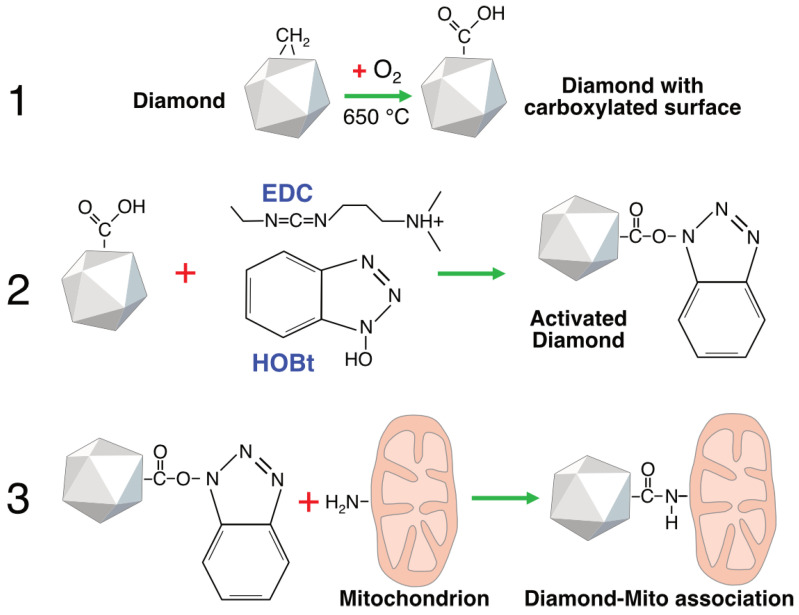
Functionalization of diamonds for binding to mitochondria via a short-chain linker. The protocol for diamond surface activation and covalent binding to isolated mitochondria involves the following steps: 1—oxidation of surface methyl groups with oxygen flow at high temperature to form carboxylic groups; 2—activation of the carboxylic groups with 1-ethyl-3-(3-dimethylaminopropyl) carbodiimide hydrochloride (EDC) and 1-hydroxybenzotriazole (HOBt); 3—linking of activated diamonds to amino groups of the proteins of outer mitochondrial membranes.

To establish a stable diamond–mitochondrion contact, we employed short-chain linkers that connect the diamond surface with proteins of the mitochondrial outer membrane. HPHT microdiamonds obtained from adamantane possess hydrogen-terminated surfaces (–CH_x_) groups after synthesis, which makes them relatively hydrophobic and chemically inert. For this reason, the particles were oxidized to generate carboxyl, carbonyl, and hydroxyl groups on their surfaces (Figure 2). In the next step, surface activation of the diamond particles was performed by converting carboxyl groups into highly reactive benzotriazolyl esters. In the final step, an amide bond was formed between the activated carboxyl groups on the diamond surface and the amino groups of mitochondrial proteins via co-incubation (see Section 4).

**Figure 3:**
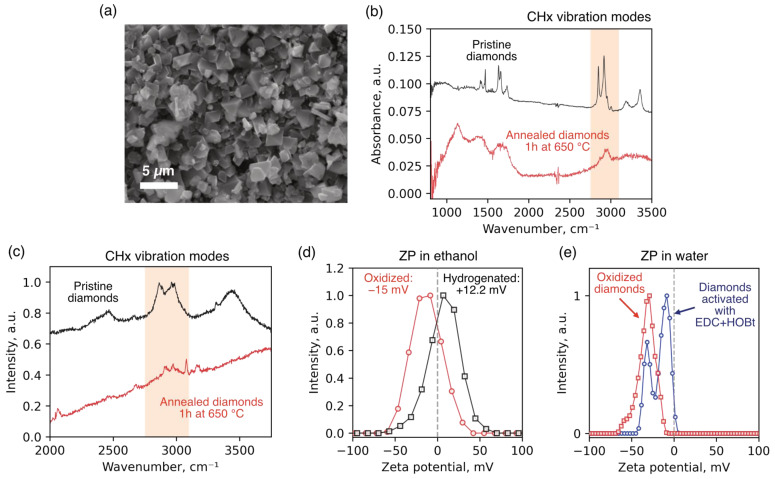
Characterization of diamond particles: (**a**)—Scanning electron images of diamond particles in powder. The particle sizes range from 200 nm to 4 µm. (**b**)—IR absorption spectra (presented in units of 1 − T, where T is transmittance) measured at room temperature for the initial (black solid) and annealed at 650 °C (red solid) samples. The spectra are corrected for the scattering losses at 6000 cm^−1^, which are assumed to be constant in the range 1000–6000 cm^−1^. Structured bands within 2800–3000 cm^−1^ are related to CH_x_ vibration modes. (**c**)—Room temperature Raman spectra of the pristine (black solid) and annealed (red solid) diamond powders. (**d**)—Zeta potential of diamond particles dispersed in ethanol before (black solid) and after annealing at 650 °C during 1 h. There is a sign change in the value of zeta potential after the surface of diamonds is oxidized. (**e**)—Zeta potential of oxidized diamond particles dispersed in water before (blue solid) and after (red solid) surface activation.

Oxidation of the diamond surface was carried out by thermal annealing in air. Prior to annealing, diamond particles were dispersed onto a silicon substrate to ensure uniform oxygen access, and then subjected to annealing in a Linkam TS1500 chamber (Linkam Scientific, Redhill, UK) at 650 °C for 60 min. The efficiency of hydrogen substitution with oxygen-containing groups was monitored using Raman spectroscopy, FTIR spectroscopy, and zeta potential measurements. In the Raman spectra recorded before and after annealing, a significant decrease in the intensity of bands corresponding to CH_x_ group vibrations was observed in the range of 2835–2950 cm^−1^ (Figure 3c), indicating effective removal of hydrogen from the surface. A similar trend was observed in the FTIR spectra (Figure 3b), where the intensity of C–H stretching vibration bands was substantially reduced after annealing. Changes in the zeta potential of the diamond particles in ethanol further confirmed the chemical modification of the surface (Figure 3d). Before annealing, the particles exhibited a positive potential (+12.2 mV), characteristic of hydrogen-terminated surfaces. After oxidation, the zeta potential shifted into the negative range (−15 mV), indicating the appearance of negatively charged surface groups. We also identified a reduction in the absolute value of the zeta potential in diamond particles with activated surfaces (Figure 3e). The observation of two peaks in the ZP distribution suggests that a certain fraction of carboxyl groups on the diamond surface did not react with EDC + HOBt and remained unactivated. This is presumably due to partial aggregation of the particles during the activation procedure, which limited the accessibility of EDC and HOBt reagents to the surface.

**Figure 4:**
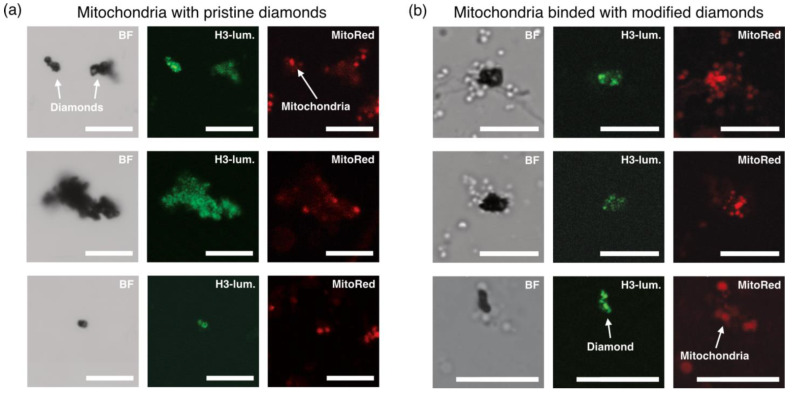
Confocal microscopy of mitochondria incubated with (**a**) non-activated diamond particles with oxidized surfaces and (**b**) EDC + HOBt-activated diamond particles. In both panels, the first column shows bright-field images; the second column displays luminescence in the 495–550 nm range, overlapping with the fluorescence signal of H3 centers in diamond; and the third column shows luminescence in the 550–720 nm range, overlapping with the fluorescence signal of MitoTracker Red. The scale bar corresponds to 10 µm.

The degree of association between diamonds with carboxylated (oxidized) and activated surfaces and mitochondria was monitored using a confocal microscope. As shown in Figure 4, co-incubation of non-activated diamonds with mitochondria followed by dilution of the suspension did not result in the formation of stable associations (Figure 4a). In contrast, incubation of mitochondria with activated diamonds led to the formation of relatively stable complexes, in which mitochondria could cover a substantial portion of the diamond surface (Figure 4b). These complexes exhibited considerable mechanical stability, as they withstood perturbations of the incubation medium during the addition of substrates and inhibitors, as well as gentle mixing. In some cases, and in specific imaging projections, mitochondrial coverage of the diamonds appeared to be two- or multilayered. This effect is presumably due to incomplete removal of EDC + HOBt from the incubation medium. We analyzed temperature changes in both “monolayer” and “multilayer” complexes.

**Figure 5:**
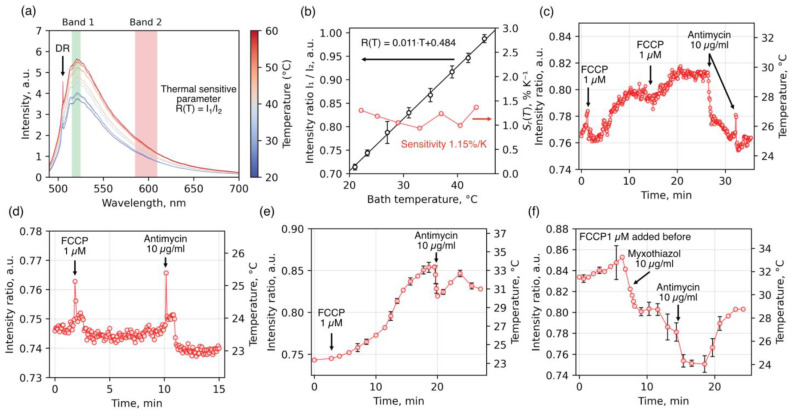
Measurement of mitochondrial temperature using a novel ratiometric approach based on N_2_V-centers luminescence in diamond particles. (**a**)—Luminescence spectra of N_2_V centers recorded at temperatures ranging from 22 °C to 70 °C, with 5 °C increments; (**b**)—Temperature dependence of the ratiometric signal defined as the ratio of integrated N_2_V luminescence intensities in the spectral ranges of 515–525 nm and 585–610 nm. (**c**–**f**)—Representative temperature traces from diamond particles coated with mitochondria in different configurations: mono-/bilayer coverage (**c**), partial coverage (**d**), and multilayer coverage (**e**,**f**), during sequential addition of an uncoupler and a respiratory chain inhibitor at the indicated final concentrations. In panel (**f**), FCCP was added 10 min prior to the start of the measurement. Representative traces from more than 35 independent recordings are shown. Representative single-particle traces chosen from more than 35 independent recordings are shown; no averaging or smoothing was applied. Fluctuations reflect measurement uncertainty at the indicated temporal resolution.

Temperature measurements of isolated mitochondria were performed using luminescent diamond microparticles containing N_2_V (or H3) color centers. These centers are characterized by bright luminescence in the visible range, including a narrow zero-phonon line (ZPL) at a wavelength of 504 nm and a broad phonon sideband extending across the entire visible and part of the near-infrared spectrum (<800 nm) (Figure 5a). To study the temperature dependence of luminescence from H3 centers, luminescence spectra of diamond particles were measured in an aqueous environment deposited on a home-built heating stage, whose temperature was varied in steps of 3–4 °C over a range of 22–50 °C (see Section 4). All measurements were performed under laser excitation at a wavelength of 473 nm and a power of 30 μW after objective. Figure 5a presents the H3 luminescence spectra. At lower temperatures, transitions that conserve the phonon state dominate, resulting in a pronounced ZPL at 504 nm. Upon heating, the intensity increases substantially in both the green and red regions of the phonon sideband, while the contribution of the ZPL near 504 nm decreases. Thus, the intensity of the H3 phonon sideband can be used as a temperature-sensitive parameter. However, to avoid artifact-induced intensity fluctuations caused by changes in the optical path (refraction/reflection) upon the addition of alcohol- or DMSO-based agents, we employed a ratiometric approach. This method utilizes the linearly increasing temperature-dependent change in the ratio $R=\frac{I_{1}}{I_{2}}$, where $I_{1}$ and $I_{2}$ are the integrated intensities in the green and red spectral regions, respectively (Figure 5b). The ratio of integrated intensities in the red and green spectral regions can be considered a reliable thermometric parameter, as it directly reflects the thermodynamic redistribution of optical transition probabilities. The relative sensitivity of the thermometric parameter R, defined as the derivative of the intensity ratio with respect to temperature, was calculated (Figure 5b, red solid line). Within the physiological temperature range, it remained constant at approximately 1.15%/°C.

To assess the potential influence of external physicochemical parameters on H3-center luminescence, we examined the effects of pH and redox-modulating agents on the ratiometric signal. Specifically, control measurements confirmed that H3 luminescence remains stable across a wide pH range (Figure S2a). Additionally, no significant changes were observed in the fluorescence spectrum or intensity of H3 centers following the addition of FCCP or antimycin A in the absence of mitochondria (Figure S2b), suggesting high inertness of diamonds with respect to incubation conditions applied in our experiments.

Using a ratiometric thermometry strategy based on H3 centers in diamonds, we measured the temperature of mitochondria isolated from rat liver during the sequential addition of an oxidative phosphorylation uncoupler and inhibition of the respiratory chain (Figure 5c–f and Figure S3). To prevent spontaneous Ca^2+^-dependent uncoupling, the incubation medium contained 1 mM EGTA and respiratory substrates (5 mM glutamate and 5 mM malate). In preliminary experiments, we did not detect any temperature increase in coupled mitochondria following the addition of respiratory substrates. This may be due either to the insufficient sensitivity of the diamond thermometer or to the presence of significant endogenous substrate reserves in liver mitochondria. Figure 5c,d, and Figure 5e,f show the temperature changes of the diamond thermometer when covered by mono-/bilayer, partial, and multilayer mitochondrial coatings, respectively. As shown in the figure, the uncoupler induced a relatively rapid temperature increase in diamonds with mono- or bilayer mitochondrial coatings (1.5–3 °C over ~5–7 min), reaching a plateau. Subsequent addition of the complex III inhibitor antimycin A caused rapid cooling of the detector (~4.5 °C over 4–5 min). When the diamond was only partially coated with mitochondria (panel D), uncoupler-induced heating was less pronounced, occurred more slowly, and showed a shorter response time to inhibitor addition. In associations with multilayer mitochondrial coatings (Figure 5e,f), we observed relatively larger temperature changes in response to inhibitor addition (~10 °C), with a longer time to reach plateau (~20 min). Furthermore, the effect of inhibitors in these multilayer associations was less pronounced or occurred in a stepwise manner, likely reflecting diffusion limitations for substrates, uncoupler, and inhibitors within the multilayered mitochondrial aggregates. These observations suggest that restricted heat dissipation from mitochondria into the surrounding environment is a key factor determining the extent of local mitochondrial heating and the corresponding temperature response of the associated diamond detector during substrate oxidation.

## 3. Discussion

In recent years, there has been ongoing debate in the literature regarding the existence of stable subcellular temperature gradients. Multiple studies have reported local temperature elevations near mitochondria by several, or even tens of, degrees Celsius. However, interpretation of these findings is complicated by the environmental sensitivity of fluorescent thermosensors and by methodological artifacts.

In the present study, we aimed to eliminate one of the key sources of systematic error in diamond-based thermometry—namely, the unstable thermal contact between the thermometer and mitochondria. To address this, diamond microparticles were chemically linked to the outer mitochondrial membrane via a short-chain linker, which significantly increased both the area and density of thermal contact and stabilized the geometry of heat transfer (Figure 2, Figure 3 and Figure 4). On this platform, we recorded reproducible changes in the ratiometric signal of H3 centers, corresponding to local temperature shifts of ~0.5–10 °C during oxidative phosphorylation uncoupling followed by inhibition of electron transport (Figure 5). This raises the question of how well the measured values correspond to temperature fluctuations that could occur under physiological conditions.

It is well established that the maximal respiration rate of liver mitochondria—under conditions of uncoupled respiration and oxidative phosphorylation and in the presence of substrates generating NADH for complex I of the respiratory chain—is close to 100 nmol/min/mg protein [2]. Oxidation of 1 mole of NADH in the respiratory chain results in the storage of approximately 91,600 J of energy in the form of ATP, with an average process efficiency of about 42% [35]. Since the reduction of one molecule of oxygen to water requires two molecules of NADH, the total heat production from the reduction of 1 mole of O_2_, in the absence of ATP generation, is approximately 436,200 J [36], which corresponds to ~727 μW/mg protein. It is known that the density of rat liver mitochondria does not exceed 1.1 g/cm^3^ [37,38]. Proteins and fatty acids in the solid state have densities of approximately 1.4 and 1.26–1.5 g/cm^3^, respectively [39]. Considering that a significant fraction of inner membrane lipids is composed of cardiolipin—which makes the membrane less dense and more fluid—a density of 1.2 g/cm^3^ can be used for mitochondrial lipids in rough estimations. Given the typical dry weight ratio of proteins to lipids in mitochondria is 60–70% to 30–40%, the approximate mass fractions in hydrated mitochondria can be estimated as ~20–25% protein, 10–13% lipids, and 65–67% aqueous phase. Thus, an organelle with a diameter of 1 μm, a volume of 0.53 μm^3^, and a mass of 0.58 pg would contain approximately 0.12 pg of protein. Therefore, one milligram of mitochondrial protein corresponds to approximately 8.3·10^9^ mitochondria. This allows for the calculation of the energy production rate of a single mitochondrion: ~8.7 × 10^−14^ W in the uncoupled state in the presence of complex I substrates (excluding heat generated during the Krebs cycle and from substrate transport across membranes).

Assuming that the specific heat capacity of anhydrous protein and lipid preparations is approximately 1.7 J/g·K, the specific heat capacity of mitochondria can be estimated at about 3.3 J/g·K, or 1.92 × 10^−12^ J/K per mitochondrion. Accordingly, an uncoupled mitochondrion would be able to raise its own temperature by 1 °C in approximately 22 s. A mitochondrion in respiratory states V2 and V3 would heat itself by 1 °C over a period roughly 10 and 2 times longer, respectively. If no heat is dissipated, a mitochondrion would heat up by 30 °C in 11 min, and by 60 °C in 22 min—unless enzyme inactivation in the Krebs cycle or respiratory chain complexes occurs earlier. Given that mitochondria are capable of functioning normally for days or even weeks (including in thermogenic tissues) [40], it is clear that heat dissipation must be sufficiently efficient.

Energetically, this scenario appears plausible at first glance. The energy released by a small aggregate of 10–12 mitochondria during uncoupling is sufficient to heat an ideal, closed “diamond–mitochondria” system to the temperatures observed in our experiments. However, when heat transfer is described in a real aqueous environment, the basic Fourier law immediately reveals a discrepancy between calculation and observation. In steady-state conditions, for a sphere of radius r, and with the thermal conductivity of water $k_{w}\approx 0.6$ W·m^−1^·K^−1^, the temperature increase at the surface is given by $∆T=\frac{P}{4\pi k_{w}r}$. Upper estimates of the power output of a single mitochondrion in the uncoupled state are on the order of ~10^−13^. If a micron-sized diamond is coated with approximately 10–12 mitochondria, the total power delivered to the “mitochondria–diamond” interface may reach ~1 pW. Substituting r=0.5 µm and P≈1 pW, we obtain a temperature rise of $∆T\approx 2.6\times 10^{-7}$ K—a value 7–8 orders of magnitude lower than that measured experimentally.

Let us consider our system through the lens of three thermally resistive layers connected in series: the diamond $R_{diamond}$, the mitochondrial “shell” $R_{mito}$, and the aqueous medium $R_{water}$. We hypothesize that the observed temperature increase may be attributed to the heat-insulating effect of the mitochondrial layer. Let us assume both the size of the diamond particle and the thickness of the insulating mitochondrial layer to be 1 μm, which is in reasonable agreement with the experimental geometry. Diamond possesses extremely high thermal conductivity (up to 10^4^ W·m^−1^·K^−1^), and therefore has a very low thermal resistance, on the order of $R_{diamond}∼10$ K/W. In contrast, the thermal resistance of the surrounding aqueous medium is approximately $R_{water}∼10^{5}$ K/W. The dominant term in this system is the mitochondrial layer, whose resistance can be expressed as $R_{mito}=\frac{\Delta r}{4\pi r_{diamond}r_{mito}k_{mito}}$. Using Fourier’s law in the form $∆T=P{\cdot R}_{total}$, we can estimate the thermal conductivity $k_{mito}$ required for the mitochondrial layer to raise the diamond particle’s temperature by 1 °C. A quick calculation yields a value in the range of 10^−6^–10^−7^, which is comparable to the thermal conductivity of high vacuum. Clearly, such a low conductivity is not physically plausible for biological material. Thus, the so-called “10^5^ paradox” first outlined by Baffou et al. [41] approximately a decade ago remains unresolved. Although our temperature measurement method is not entirely free from potential artifacts (such as optical heating of the particles or sensitivity to electric fields), the resolution of this paradox may lie in the mechanism of nanoscale heat transport—particularly pronounced during heat transfer across multilayer mitochondrial membranes.

One might hypothesize that the heating of diamond thermometers has an artifactual origin—such as optical heating by laser irradiation or an effect of local electric fields. However, the fact that this effect appears only upon the addition of an uncoupler of oxidative phosphorylation and is eliminated by the subsequent addition of respiratory chain inhibitors strongly suggests that such an explanation is insufficient. Moreover, our control experiments in the absence of mitochondria demonstrated no detectable changes in ratiometric signal upon addition of these agents (Figure S2a,b), as well as different pH.

Returning to the question of whether the observed mitochondrial temperature fluctuations are physiologically relevant, it can be stated unequivocally that the formation of multilayered mitochondrial aggregates in living cells is implausible. Nevertheless, heat dissipation from mitochondria may be impeded by other intracellular structures, including the membranes of the endoplasmic and sarcoplasmic reticulum, the Golgi apparatus, intracellular vesicles, lipid droplets, protein networks, and carbohydrate or nucleic acid polymers.

## 4. Materials and Methods

### 4.1. Materials

Antimycin A (AntA) (A8674), bovine serum albumin (BSA) (A2153), carbonyl cyanide p-(trifluoromethoxy)phenylhydrazone (FCCP) (C2920), cyclosporin A (CsA) (30024), 4-(2-hydroxyethyl)piperazine1-ethanesulfonic acid (HEPES) (H3375), glutamate (G1501), malate (8.20872), mannitol (M4125), myxothiazol (Myx) (T5580), rotenone (Rot) (R8875), succinate (W327700), sucrose (S7903), and Trizma Base (93352) were purchased from Merck KGaA (Darmstadt, Germany). Ethylene glycol-bis(2-aminoethylether)-N,N,N0,N0-tetraacetic acid (EGTA) (A0878,0025) was obtained from PanReac AppliChem ITW Reagents (Darmstadt, Germany). Mito Tracker^TM^ Red CMX ROS (M7512) was from Thermo Fisher Scientific Inc, Waltham, MA, USA. Other chemicals were of analytical grade and were purchased from local suppliers.

### 4.2. Isolation and Staining of Rat Liver Mitochondria

All manipulations with animals were conducted in accordance with the Helsinki Declaration of 1975 (revised in 1983), the national regulations for the care and use of laboratory animals, and protocol No. 5/2025 dated 3 March 2025, approved by the Commission on Biological Safety and Bioethics at Institute of Theoretical and Experimental Biophysics of Russian Academy of Sciences. Rat liver mitochondria were isolated by the standard method of Johnson and Lardy [42] modified as follows [43]. The homogenization medium consisted of 220 mM mannitol, 70 mM sucrose, 10 mM HEPES (pH adjusted to 7.3 with KOH), 1 mM EGTA, and 0.05% BSA. The homogenate was centrifuged at 600× *g* for 10 min at 4 °C, and the supernatant fraction was then centrifuged at 7000× *g* for 15 min to sediment mitochondria. The mitochondria were washed two times in the same medium without EGTA and BSA. The final pellets were resuspended in EGTA- and BSA-free medium (~70 mg protein/mL final concentration). The mitochondrial protein concentration was determined using the Biuret method with BSA as a standard.

Measurements were performed at room temperature (23 °C) in the standard KCl-based medium (125 mM KCl, 25 mM HEPES (pH 7.3 adjusted with KOH), 2 mM MgCl_2_, 0.5 mM KH_2_PO_4_, and 1 mM EGTA) or sucrose-mannitol-based medium (210 mM mannitol, 70 mM sucrose, 10 mM HEPES (pH 7.3 adjusted with KOH), 2 mM MgCl_2_, 2 mM KH_2_PO_4_, and 1 mM EGTA) supplemented with substrates and inhibitors as specified in figure legends.

### 4.3. Assessment of Mitochondrial Quality

Mitochondrial quality was assessed by the determination of respiratory control coefficient. The respiratory control coefficient was assessed by measuring the rates of oxygen consumption by mitochondria (1 mg prot./mL) before, during, and after the phosphorylation of 200 nmols of ADP (200 µM final concentration) in the presence of respiratory substrates. The rate of mitochondrial respiration was evaluated using the Oroboros Oxygraph-2k (Innsbruck, Austria) in KCl-based medium [44]. In this study, the respiratory control coefficient of fresh mitochondria with glutamate and malate was about 6.

### 4.4. Synthesis of Diamonds

Diamond powders were produced using the high-pressure high-temperature (HPHT) technique from a mixture of adamantane (C_10_H_16_, Sigma-Aldrich, St. Louis, MO, USA, 99% purity) and detonation nanodiamonds (DND, Adamas Nanotechnologies Inc., Raleigh, NC, USA, average size 3–4 nm) containing ~1% nitrogen impurity. The samples were synthesized at a pressure of ~7.5 GPa and a temperature range of 1500–1600 °C for 20 s. For sample preparation, the following weight ratios of adamantane to DND were used 1:2 and 300:1. The nitrogen impurity in DND was the source of two nitrogen atom-vacancy (NVN or H3) fluorescent centers formed in the 1:2 diamonds during HPHT synthesis. Tetrakis(trimethylsilyl)silane (C_12_H_36_Si_5_, Sigma-Aldrich, St. Louis, MO, USA, >97%) was added to the initial mixture to form SiV centers in the 300:1 diamonds.

The microdiamonds used in the temperature measurements ranged from 200 nm to 4 µm in size, as confirmed by SEM (Figure 3a). Two types of HPHT-synthesized diamonds were employed: (1) diamonds enriched with nitrogen-vacancy-nitrogen (NVN, or H3) centers, produced from a 1:2 weight ratio of adamantane to DND, where DND contains ~1% nitrogen impurity necessary for the formation of H3 centers, and (2) diamonds containing SiV centers, obtained from a 300:1 ratio in the presence of tetrakis(trimethylsilyl)silane as a silicon source stimulated the SiV formation. Although the absolute concentration of luminescent centers in the bulk was not quantified, thermometric measurements were performed using individual particles exhibiting strong photoluminescence from the corresponding color center, verified spectrally prior to experiments.

### 4.5. Activation of Diamonds and Their Binding to Mitochondria

CH_x_ groups on the diamond surfaces were oxidized to carboxylic groups via annealing on the silicone base in the Linkam TS1500 chamber at 650 °C for 60 min [45]. Carboxylated diamonds (~100 µg) were placed in 50 mM MES buffer (pH 5.0) supplemented with 4% 1-ethyl-3-(3-dimethylaminopropyl) carbodiimide hydrochloride (EDC) and mixed with equal volume of 10% 1-hydroxybenzotriazole (HOBt) dissolved in dimethylacetamide [46]. The suspension was incubated for 20 min at room temperature with two 5 min cycles of ultrasound treatment in a water bath. The suspension of activated diamonds was diluted with 3 volumes of MES buffer and centrifuged at 15,000× *g* for 5 min. The sediment was washed once with the same buffer and dried with a pipette. Mitochondria (20 mg prot./mL) were placed in high-pH Bicine buffer (125 mM KCl, 50 mM Bicine (pH 8.25 adjusted with KOH), 2 mM MgCl_2_, 0.5 mM KH_2_PO_4_, and 1 mM EGTA), immediately mixed with activated diamonds, and incubated at room temperature in the dark for 30 min. Then, mitochondria were gently dissolved with the standard KCl-based medium and kept on ice until use within <2 h.

### 4.6. Confocal Fluorescent Microscopy

The efficiency of mitochondrial binding to activated diamonds was assessed by confocal fluorescent microscopy. Mitochondria (5 mg prot./mL) were placed in the isolation medium supplemented with 1 mM EGTA, 5 mM glutamate, 5 mM malate, and 1 µM MitoTracker™ Red CMXRos (Thermo Fisher Scientific Inc., Waltham, MA, USA) without BSA. After 10 min incubation in the dark at room temperature, mitochondria were sedimented (at 9000 g for 7 min), resuspended in the Bicine buffer, and bound to the activated diamonds. The aggregates of diamonds with stained mitochondria were examined by a Leica TCS SP5 confocal microscope (Leica, Wetzlar, Germany) using 543 nm excitation lasers and 560–664 nm emission filter.

### 4.7. Raman Spectroscopy

Raman measurements were performed at room temperature using a LabRAM HR800 spectrometer (HORIBA, Palaiseau, France) equipped with a 473 nm diode laser. The excitation laser beam (1 mW power reaching the sample) was focused through a 100× Olympus objective (NA = 0.95). And the scattered light was collected in the backscattering geometry.

### 4.8. FTIR Measurements

FTIR spectra were measured using a Bruker Vertex 70 v spectrometer (Bruker Optics GmbH, Ettlingen, Germany) with a Hyperion microscope.

### 4.9. Temperature Recordings

Local temperature measurements were carried out using two different optical approaches on a home-built confocal microscope (Figure S1). The first method is based on tracking the spectral shift of the zero-phonon line (ZPL) of the photoluminescence from SiV centers in diamond particles (sample with adamantane to DND ratio 300:1), which is sensitive to temperature changes. This method has been described in detail in our previous work [30,31,47]. The second approach relies on the temperature sensitivity of luminescence from H3 centers in diamond (sample with adamantane to DND ratio 1:2). As the temperature-dependent parameter, a ratiometric ratio of the integrated intensities in the spectral ranges of 515–525 nm (I_1_) and 585–610 nm (I_2_) was used. For H3-based diamond thermometers, the calibration curve as a function of temperature was measured using a homemade heating stage, capable of controlled heating in the range of 20 to 50 °C in an aqueous environment. Temperature measurements of isolated mitochondria were performed with a temporal resolution ranging from 1 to 5 s.

## 5. Conclusions

In this work, we developed a hybrid thermometric platform based on chemically functionalized diamond microparticles containing H3 luminescent centers, covalently bound to the outer membrane of isolated mitochondria. This approach provided a robust and reproducible thermal interface that minimized heat dissipation through the surrounding medium. Using a ratiometric readout from the H3 centers, we detected reproducible local temperature elevations of 0.5–10 °C upon uncoupling of oxidative phosphorylation and inhibition of the electron transport chain, with the magnitude depending on the extent of mitochondrial coverage.

The calculations and experiments presented here demonstrate that the observed temperature elevations near mitochondria cannot be explained solely by thermal effects in a stationary aqueous medium. Their interpretation requires consideration not only of the contact geometry between the diamond and the organelle but also of the peculiarities of nanoscale heat transfer, where the applicability of the classical Fourier law may be limited. Consequently, further investigation of heat transfer in such hierarchical, multilayered, and dynamic systems appears to be key to understanding the origin of the observed local overheating and may shed light on the fundamental mechanisms of mitochondrial thermogenesis.

## Data Availability

The original contributions presented in this study are included in the article/Supplementary Materials. Further inquiries can be directed to the corresponding authors.

## Supplementary Materials

The supporting information can be downloaded at: https://www.mdpi.com/article/10.3390/ijms262311395/s1.
