# Supplementary material for: Chemically Anchored Diamond with H3 Centers for Ratiometric Measurement of Isolated Mitochondria Temperature
Source: Int J Mol Sci. 2025 Nov 25;26(23):11395. doi: 10.3390/ijms262311395 (PMC12692260; doi:10.3390/ijms262311395)
Supplement: Supplementary file 1 [file ijms-26-11395-s001.zip › ijms-3985601-supplementary.pdf]

Supplementary Information

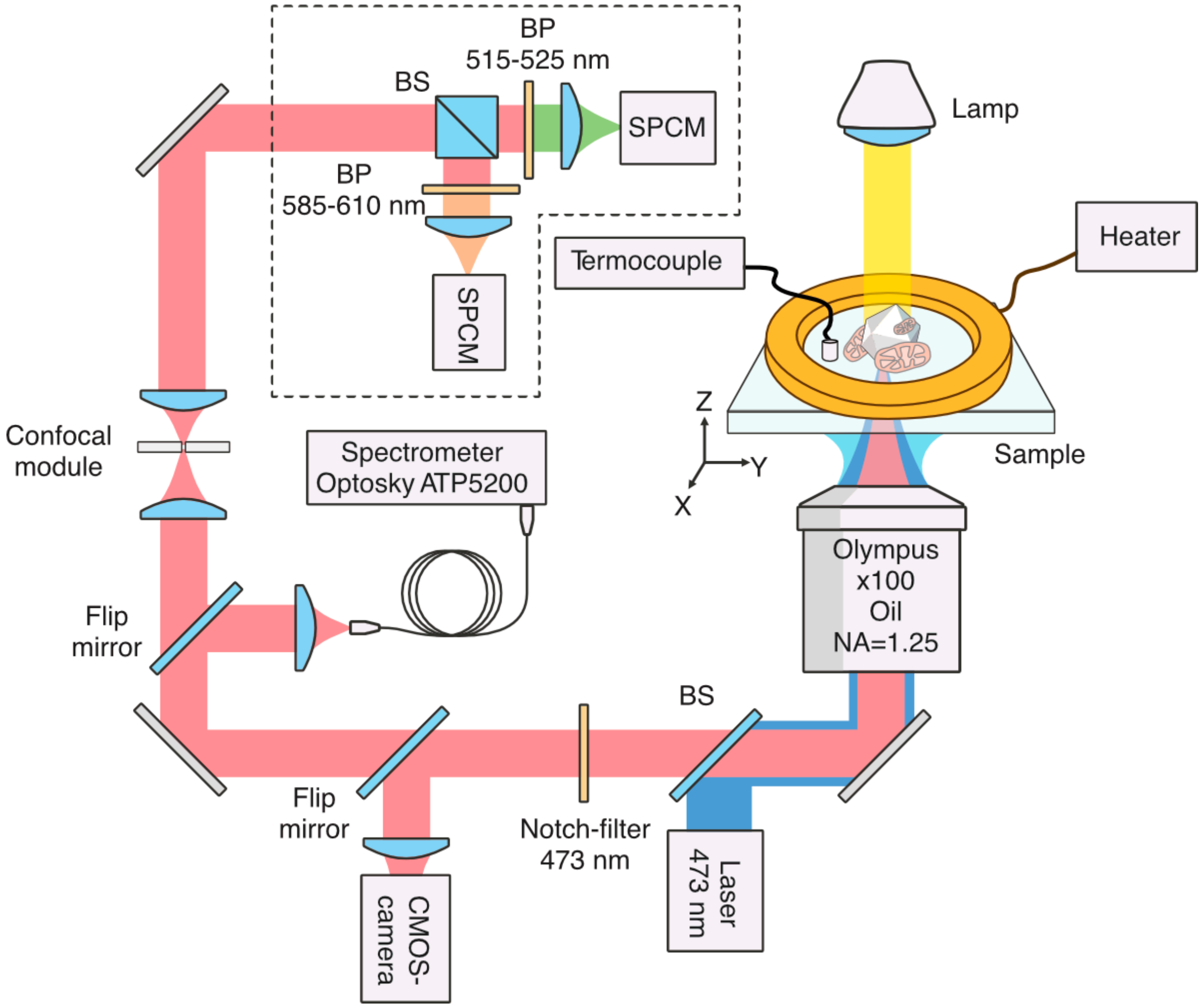

Figure S1. Schematic of the experimental setup used for temperature measurements based on H3-center luminescence in diamond microparticles.

The luminescent properties of NDs were examined in a custom-made confocal microscope (**Fig. S1**), equipped with a high-aperture immersive objective Olympus Plan N x100/NA=1.25 oil and a piezoelectric stage (piezosystem jena TRITOR 100), enabling precise 3D scanning with nanoscale resolution. The handmade heater with thermocouple was used to control the temperature of the sample. Optical images of the nanodiamonds were captured by a high-resolution CMOS camera under wide-field illumination. To excite the luminescence of NDs laser source emitting at 473 nm (Cobolt 05-01) was used. To eliminate reflected laser light, notch filter 473±5 nm was included in the optical path. The spectra of NDs were recorded with spectrometer Optosky ATP5200 with 100 μm slit at the entrance. The pin hole was used to decrease illumination from the volume. The Hanbury-Brown-Twiss

interferometer with two detectors (Excelitas SPCM–AQRH–14–FC) together with Time Tagger 20 (Swabian Instruments) were employed. Band pass (BP) filter @515-525 nm and BP filter @585-610 nm before detectors were used to measure time trace for each channel.

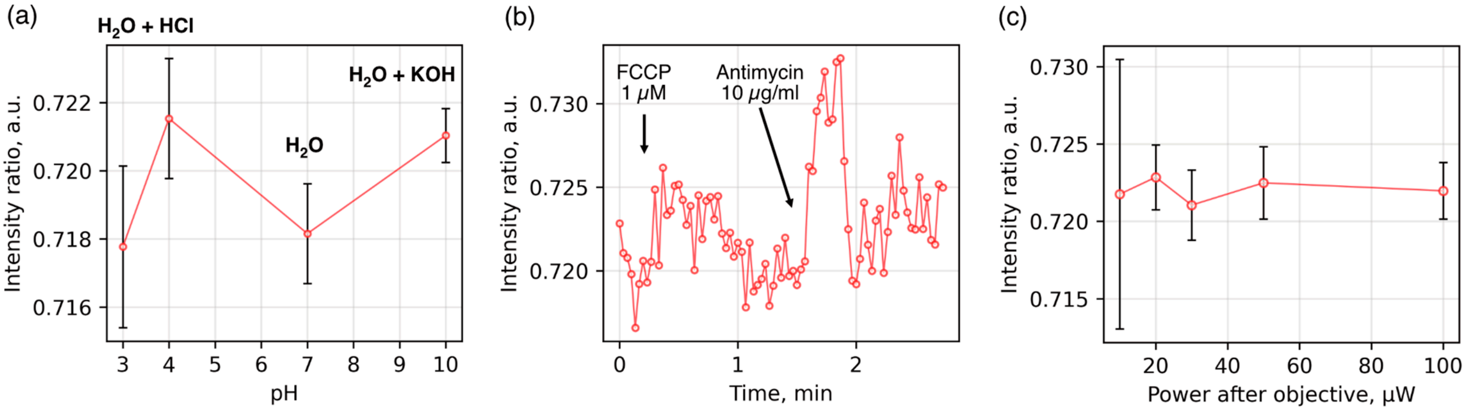

Figure S2. Effects of pH and redox-modulating agents on the ratiometric signal of H3-luminescent signal.

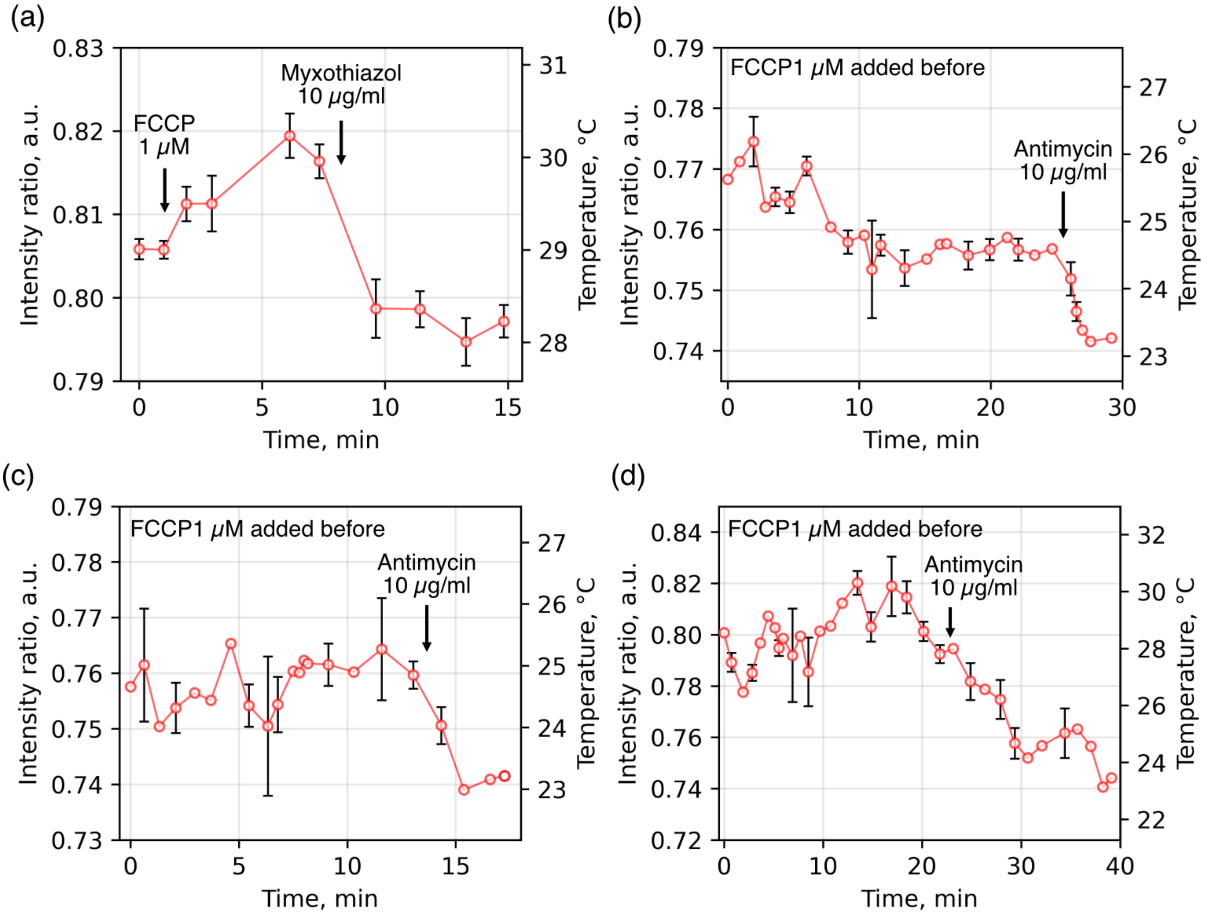

Figure S3. Measurement of mitochondrial temperature using a novel ratiometric approach based on H3-center luminescence in diamond particles.
